# Vagus Nerve Stimulation: A Potential Adjunct Therapy for COVID-19

**DOI:** 10.3389/fmed.2021.625836

**Published:** 2021-05-07

**Authors:** Eric Azabou, Guillaume Bao, Rania Bounab, Nicholas Heming, Djillali Annane

**Affiliations:** ^1^Clinical Neurophysiology and Neuromodulation Unit, Departments of Physiology and Critical Care Medicine, Raymond Poincaré Hospital, Assistance Publique- Hôpitaux de Paris, Inserm UMR 1173, Infection and Inflammation (2I), University of Versailles Saint-Quentin en Yvelines (UVSQ), Paris-Saclay University, Paris, France; ^2^General Intensive Care Unit - Assistance Publique Hôpitaux de Paris, Raymond Poincaré Hospital, Assistance Publique- Hôpitaux de Paris, Inserm UMR 1173, Infection and Inflammation (2I), University of Versailles Saint-Quentin en Yvelines (UVSQ), Paris-Saclay University, Paris, France

**Keywords:** COVID-19, cytokine storm, inflammation, non-drug therapy, vagus nerve stimulation, neuromodulation, outcome

## Abstract

The novel severe acute respiratory syndrome coronavirus 2 (SARS-CoV-2) causes coronavirus disease 2019 (COVID-19) through excessive end organ inflammation. Despite improved understanding of the pathophysiology, management, and the great efforts worldwide to produce effective drugs, death rates of COVID-19 patients remain unacceptably high, and effective treatment is unfortunately lacking. Pharmacological strategies aimed at modulating inflammation in COVID-19 are being evaluated worldwide. Several drug therapies targeting this excessive inflammation, such as tocilizumab, an interleukin (IL)-6 inhibitor, corticosteroids, programmed cell death protein (PD)-1/PD-L1 checkpoint inhibition, cytokine-adsorption devices, and intravenous immunoglobulin have been identified as potentially useful and reliable approaches to counteract the cytokine storm. However, little attention is currently paid for non-drug therapeutic strategies targeting inflammatory and immunological processes that may be useful for reducing COVID-19-induced complications and improving patient outcome. Vagus nerve stimulation attenuates inflammation both in experimental models and preliminary data in human. Modulating the activity of cholinergic anti-inflammatory pathways (CAPs) described by the group of KJ Tracey has indeed become an important target of therapeutic research strategies for inflammatory diseases and sepsis. Non-invasive transcutaneous vagal nerve stimulation (t-VNS), as a non-pharmacological adjuvant, may help reduce the burden of COVID-19 and deserve to be investigated. VNS as an adjunct therapy in COVID-19 patients should be investigated in clinical trials. Two clinical trials on this topic are currently underway (NCT04382391 and NCT04368156). The results of these trials will be informative, but additional larger studies are needed.

## Introduction

The coronavirus disease 2019 (COVID-19) pandemic caused by the novel severe acute respiratory syndrome coronavirus 2 (SARS-CoV-2) faced currently worldwide includes in its pathophysiology an excessive inflammatory phase called “cytokine storm” that is closely linked to its high mortality (1, 2). During sepsis, the host response to a pathogen is mediated by the interaction between pathogen-associated molecular pattern and their receptors located on innate immune cells (3). This interaction leads to activation of the innate immune cell, release of inflammatory cytokines, and recruitment of further cells of the immune system (4). When this immune response is exaggerated, excessive inflammation may lead to end tissue damage. All major organs may be affected during sepsis including altered hypothalamic–pituitary–adrenal and altered cardiovascular responses (5, 6). Disruption of the hypothalamic–pituitary–adrenal axis may translate in patients with sepsis into cardiovascular and organ dysfunction and an increase in the risk of death (5, 6). Impaired heart rate variability and high concentrations of circulating catecholamines and impaired sympathetic modulation are common findings of septic and septic shock patients, reflecting dysfunction of the medullary autonomic centers (7) and suggesting that central autonomic regulatory impairment contributes to circulatory failure (8–10). Clinical patterns concordant with these hypotheses have been documented in COVID-19 patients and support the hypothesis of a potential contribution of a dysfunction in autonomic tone to the cytokine release syndrome and related multiorgan damage in COVID-19 (11–17).

Specific treatment for COVID-19 is unfortunately lacking (18, 19). However, given the high mortality rate and economic damage to date, great efforts are being made worldwide to produce successful drugs (20). Particularly, pharmacological strategies that restore inflammatory control or inhibit cytokine release are being evaluated (21–23). Several drug therapies targeting this excessive inflammation, such as tocilizumab, an interleukin (IL)-6 inhibitor, corticosteroids, programmed cell death protein (PD)-1/PD-L1 checkpoint inhibition, cytokine-adsorption devices, and intravenous immunoglobulin have been identified as potentially useful and reliable approaches to counteract cytokine storm in COVID-19 patients (1, 2, 18, 24–32). Little attention is currently paid for non-drug therapeutic strategies targeting inflammatory and immunological processes that may be useful for reducing COVID-19-induced complications and improving patient outcome (33–35).

## Vagus Nerve Stimulation a Potential Adjunct Therapy in COVID-19

Modulating the activity of cholinergic anti-inflammatory pathways (CAPs) described by the group of KJ Tracey (36–44) has indeed become an important target of therapeutic research strategies for inflammatory diseases and sepsis (37, 38, 45–47). In fact, the CAP pathways innervate the spleen through the efferent vagus nerve and the splenic nerve relay and act on macrophages by transforming adrenergic stimulation into a cholinergic signal by the T cells of the spleen, which plays an anti-inflammatory effect (48).

About 80% of the vagus nerve is composed of afferent sensory fibers carrying information from the periphery to the brain (49). Within the central nervous system, the vagus primarily projects to the nucleus of the solitary tract (NTS), releasing excitatory neurotransmitters (glutamate and aspartate), inhibitory neurotransmitter (gamma-aminobutyric acid), acetyl-choline, norepinephrine, and neuropeptides implicated in signal transduction (49). In turn, the NTS has widespread efferent pathways to the parabrachial nucleus, reticular formation, basal forebrain, amygdala, hippocampus, hypothalamus, dorsal raphe, cerebellum, and spinal cord (50). NTS projections to brainstem nuclei (locus coeruleus and dorsal raphe magnus) modulate serotonin and norepinephrine release to the entire brain (51). Through efferent and afferent fibers, the vagus nerve plays a role in maintaining cardiovascular homeostasis and in modulating inflammation (52). The autonomic nervous system regulates the production of cytokines, through interactions with the hypothalamic–pituitary–adrenal axis, leading to the release of anti-inflammatory glucocorticoid hormones. Vagal efferent fibers also release acetylcholine (Ach), which, by interacting with α7-subunit-containing nicotinic receptors found in tissue macrophages, and dendritic cells, inhibit the release of proinflammatory cytokines such as tumor necrosis factor alpha (TNFα), IL-1β, IL-6, and IL-18 (36, 53). Inflammatory reflex signaling, which is enhanced by electrically stimulating the vagus nerve, significantly reduces cytokine production and attenuates disease severity in animal models of inflammatory diseases and in experimental models of sepsis (36, 54–57). Electrical stimulation of the vagus nerve attenuates inflammation in a variety of pathological conditions with little side effects (36, 58, 59). Recently, Meneses and colleagues demonstrated that vagus nerve stimulation attenuates the inflammatory response in the central nervous system induced by peripheral lipopolysaccharide challenge in rats (60). Kohoutova and colleagues recently demonstrated that vagus nerve stimulation attenuates multiple organ dysfunction in a porcine model of sepsis (61). These findings suggest that VNS could be a promising adjunctive therapy targeting inflammatory pathways in COVID-19 patients. VNS might attenuate sepsis-related inflammatory processes leading to endothelial activation, impaired microcirculation, multiorgan failure, and death. VNS may also exhibit favorable cardiovascular effects during sepsis, including antiarythmogen, decreased myocardial oxygen consumption, and improved diastole (62). Vagus nerve stimulation has a favorable safety track record. Implanted VNS devices have been used for decades to treat refractory partial-onset seizures and severe recurrent refractory depression with confirmed safety and only mild to moderate side effects that are predictable improve over time (63–65). More recently, non-invasive transcutaneous vagus nerve stimulation devices (t-VNS) have been developed and commercialized (66). Evidence from preclinical models (61, 67) as well as from several clinical reports (47, 68) is accumulating (68–72). Boezaart and Botha reported a drastic reduction of two COVID-19 patients treated with t-VNS (69). Non-invasive VNS as adjunct therapy in COVID-19 patients might alleviate organ dysfunction and improve patients' outcome. Randomized controlled studies assessing the effectiveness of non-invasive vagus nerve stimulation as adjunct therapy to current best medical practice for COVID-19 are needed (72). Two studies evaluating the efficacy of non-invasive VNS in COVID-19 patients are now on going using the gammaCore® non-invasive vagal nerve stimulation device. The gammaCore® (electroCore, Inc., Basking Ridge, NJ) is handheld and requires no surgery or implants. The device is applied by healthcare providers or patients to the skin at the neck over the vagus nerve to deliver periodic doses of VNS non-invasively. Tariq Cheema and colleagues are conducting a prospective, randomized, controlled investigation designed to assess the reduction in respiratory distress in a COVID-19 population: the SAVIORII study NCT04382391. The primary objective is to reduce initiation of mechanical ventilation in patients with COVID-19 compared to the control group. Secondary objectives are to evaluate cytokine trends/prevent cytokine storms, evaluate supplemental oxygen requirements, decrease mortality of COVID-19 patients, and delay the onset of mechanical ventilation. The second ongoing clinical trial using the same device is conducted by Carlos Tornero and colleagues NCT04368156: the SAVIOR study. The SAVIOR study is a prospective, randomized, controlled study assessing vagus nerve stimulation in COVID-19 respiratory symptoms (72). The primary outcome measures were incidence of changes in specific clinical events such as the proportion of subjects requiring mechanical ventilation, days to onset of mechanical ventilation, oxygen support requirements, O_2_ saturation, pain levels, PaO_2_/FiO_2_, coagulation, laboratory measurements related to circulating cytokines and inflammation, live discharge from the hospital, patient length of stay, mortality, need for intensive care, shortness of breath, device-related serious adverse events, and adverse events. The results of these trials will be informative, but additional, larger, studies are needed.

## Discussion

COVID-19 remains a major healthcare issue worldwide. Excessive inflammation and its end organ consequences are key elements in the pathogenesis of COVID-19-induced multiple organ dysfunction (19, 26, 32). Specific treatment for COVID-19 is unfortunately lacking. Several promising pharmacological strategies aimed at modulating inflammation in COVID-19 are being evaluated worldwide. However, little attention is currently paid for non-drug therapeutic strategies targeting inflammatory and immunological processes, which may be useful for reducing COVID-19-induced complications and improving patient outcome (33–35). Vagal neurostimulation has a wide field of therapeutic benefit for patients and should be combined with the best current medical strategies (15, 17, 69, 70). Vagus nerve stimulation attenuates inflammation both in experimental models and preliminary data in man. The development non-invasive vagal nerve stimulation (t-VNS), a non-pharmacological adjuvant, may help reduce the burden of COVID-19 and deserve to be investigated. The aim of this paper is to promote the emergence of original studies assessing non-invasive VNS as an adjuvant treatment for the management of COVID-19.

## Author Contributions

EA and DA conceived the manuscript. EA, NH, GB, RB, and DA were involved in early discussions and mapping the concepts that led to this paper and wrote the first draft of the manuscript. All authors read and critically reviewed drafts of the manuscript.

## Conflict of Interest

The authors declare that the research was conducted in the absence of any commercial or financial relationships that could be construed as a potential conflict of interest.

## References

[1] Soy M Keser G Atagunduz P Tabak F Atagunduz I Kayhan S. Cytokine storm in COVID-19: pathogenesis and overview of anti-inflammatory agents used in treatment. Clin Rheumatol. (2020) 39:2085–94. 10.1007/s10067-020-05190-5 32474885 PMC7260446

[2] Vergallo C. Infusion of HLA-matched and static magnetic field-exposed allogenic lymphocytes treating lymphocytopenia and cytokine storm syndrome: a treatment proposal for COVID-19 patients. Electromagn Biol Med. (2020) 40:11–25. 10.1080/15368378.2020.1830290 33073612

[3] Klucinski P Martirosian G. Role of cytokines and pathogen associated molecular pattern receptors in sepsis. Przegl Epidemiol. (2005) 59:695–701. 16433311

[4] Angus DC van der Poll T. Severe sepsis and septic shock. N Engl J Med. (2013) 369:2063. 10.1056/NEJMc1312359 24256390

[5] Annane D. The role of ACTH and corticosteroids for sepsis and septic shock: an update. Front Endocrinol (Lausanne). (2016) 7:70. 10.3389/fendo.2016.00070 27379022 PMC4913096

[6] da Costa LH Junior NN Catalao CH Sharshar T Chretien F da Rocha MJ. Vasopressin impairment during sepsis is associated with hypothalamic intrinsic apoptotic pathway and microglial activation. Mol Neurobiol. (2016) 54:5526–33. 10.1007/s12035-016-0094-x 27631877

[7] Aboab J Polito A Orlikowski D Sharshar T Castel M Annane D. Hydrocortisone effects on cardiovascular variability in septic shock: a spectral analysis approach. Crit Care Med. (2008) 36:1481–6. 10.1097/CCM.0b013e31816f48f2 18434902

[8] Annane D Trabold F Sharshar T Jarrin I Blanc AS Raphael JC. Inappropriate sympathetic activation at onset of septic shock: a spectral analysis approach. Am J Respir Crit Care Med. (1999) 160:458–65. 10.1164/ajrccm.160.2.9810073 10430714

[9] Annane D. Adjunctive treatment in septic shock: what's next? Presse Med. (2016) 45:105–9. 10.1016/j.lpm.2016.03.004 27085987

[10] Annane D Buisson CB Cariou A Martin C Misset B Renault A. Design and conduct of the activated protein C and corticosteroids for human septic shock (APROCCHSS) trial. Ann Intensive Care. (2016) 6:43. 10.1186/s13613-016-0165-1 27154719 PMC4859323

[11] Ghosh R Roy D Sengupta S Benito-Leon J. Autonomic dysfunction heralding acute motor axonal neuropathy in COVID-19. J Neurovirol. (2020) 26:964–6. 10.1007/s13365-020-00908-2 32918164 PMC7485423

[12] Goldstein DS. The extended autonomic system, dyshomeostasis, and COVID-19. Clin Auton Res. (2020) 30:299–315. 10.1007/s10286-020-00714-0 32700055 PMC7374073

[13] Gonzalez-Duarte A Norcliffe-Kaufmann L. Is 'happy hypoxia' in COVID-19 a disorder of autonomic interoception? A hypothesis. Clin Auton Res. (2020) 30:331–3. 10.1007/s10286-020-00715-z 32671502 PMC7362604

[14] Guaraldi P Barletta G Baschieri F Calandra-Buonaura G Provini F Cortelli P. Testing cardiovascular autonomic function in the COVID-19 era: lessons from Bologna's Autonomic Unit. Clin Auton Res. (2020) 30:325–30. 10.1007/s10286-020-00710-4 32661775 PMC7355131

[15] Figueroa JJ Cheshire WP Claydon VE Norcliffe-Kaufmann L Peltier A Singer W. Autonomic function testing in the COVID-19 pandemic: an American Autonomic Society position statement. Clin Auton Res. (2020) 30:295–7. 10.1007/s10286-020-00702-4 32529405 PMC7287407

[16] Logmin K Karam M Schichel T Harmel J Wojtecki L. Non-epileptic seizures in autonomic dysfunction as the initial symptom of COVID-19. J Neurol. (2020) 267:2490–1. 10.1007/s00415-020-09904-2 32458194 PMC7249974

[17] Chigr F Merzouki M Najimi M. Autonomic brain centers and pathophysiology of COVID-19. ACS Chem Neurosci. (2020) 11:1520–2. 10.1021/acschemneuro.0c00265 32427468

[18] Tang Y Liu J Zhang D Xu Z Ji J Wen C. Cytokine storm in COVID-19: the current evidence and treatment strategies. Front Immunol. (2020) 11:1708. 10.3389/fimmu.2020.01708 32754163 PMC7365923

[19] Wiersinga WJ Rhodes A Cheng AC Peacock SJ Prescott HC. Pathophysiology, transmission, diagnosis, and treatment of coronavirus disease 2019 (COVID-19): a review. JAMA. (2020) 324:782–93. 10.1001/jama.2020.12839 32648899

[20] Boregowda U Perisetti A Nanjappa A Gajendran M Kutti Sridharan G Goyal H. Addition of tocilizumab to the standard of care reduces mortality in severe COVID-19: a systematic review and meta-analysis. Front Med (Lausanne). (2020) 7:586221. 10.3389/fmed.2020.586221 33123544 PMC7566918

[21] Calabrese LH. Cytokine storm and the prospects for immunotherapy with COVID-19. Cleve Clin J Med. (2020) 87:389–93. 10.3949/ccjm.87a.ccc008 32393592

[22] Horowitz RI Freeman PR. Three novel prevention, diagnostic, and treatment options for COVID-19 urgently necessitating controlled randomized trials. Med Hypotheses. (2020) 143:109851. 10.1016/j.mehy.2020.109851 32534175 PMC7242962

[23] Wang J Jiang M Chen X Montaner LJ. Cytokine storm and leukocyte changes in mild versus severe SARS-CoV-2 infection: review of 3939 COVID-19 patients in China and emerging pathogenesis and therapy concepts. J Leukoc Biol. (2020) 108:17–41. 10.1002/JLB.3COVR0520-272R 32534467 PMC7323250

[24] Gonzalez-Nicolas MA Gonzalez-Guerrero C Perez-Fernandez VA Lazaro A. Cilastatin: a potential treatment strategy against COVID-19 that may decrease viral replication and protect from the cytokine storm. Clin Kidney J. (2020) 13:903–5. 10.1093/ckj/sfaa193 33117530 PMC7543365

[25] Chitturi KR Thacker S Al-Saadi MA Kassi M. Successful treatment of acute heart failure in COVID-19-induced cytokine storm with tocilizumab: a case report. Eur Heart J Case Rep. (2020) 4:1–6. 10.1093/ehjcr/ytaa188 33089052 PMC7454490

[26] Mustafa MI Abdelmoneim AH Mahmoud EM Makhawi AM. Cytokine storm in COVID-19 patients, its impact on organs and potential treatment by QTY code-designed detergent-free chemokine receptors. Mediators Inflamm. (2020) 2020:8198963. 10.21467/preprints.139 33029105 PMC7512100

[27] Bradshaw PC Seeds WA Miller AC Mahajan VR Curtis WM. COVID-19: proposing a ketone-based metabolic therapy as a treatment to blunt the cytokine storm. Oxid Med Cell Longev. (2020) 2020:6401341. 10.1155/2020/6401341 33014275 PMC7519203

[28] Langer-Gould A Smith JB Gonzales EG Castillo RD Figueroa JG Ramanathan A. Early identification of COVID-19 cytokine storm and treatment with anakinra or tocilizumab. Int J Infect Dis. (2020) 99:291–7. 10.1016/j.ijid.2020.07.081 32768693 PMC7406519

[29] Yessayan L Szamosfalvi B Napolitano L Singer B Kurabayashi K Song Y. Treatment of cytokine storm in COVID-19 patients with immunomodulatory therapy. ASAIO J. (2020) 66:1079–83. 10.1097/MAT.0000000000001239 33136592 PMC10660617

[30] Farooqi F Dhawan N Morgan R Dinh J Nedd K Yatzkan G. Treatment of severe COVID-19 with tocilizumab mitigates cytokine storm and averts mechanical ventilation during acute respiratory distress: a case report and literature review. Trop Med Infect Dis. (2020) 5:112. 10.3390/tropicalmed5030112 32635353 PMC7559384

[31] Dalamaga M Karampela I Mantzoros CS. Commentary: Phosphodiesterase 4 inhibitors as potential adjunct treatment targeting the cytokine storm in COVID-19. Metabolism. (2020) 109:154282. 10.1016/j.metabol.2020.154282 32497535 PMC7263254

[32] Saha A Sharma AR Bhattacharya M Sharma G Lee SS Chakraborty C. Tocilizumab: a therapeutic option for the treatment of cytokine storm syndrome in COVID-19. Arch Med Res. (2020) 51:595–7. 10.1016/j.arcmed.2020.05.009 32482373 PMC7241374

[33] Azabou E Bao G Heming N Bounab R Moine P Chevallier S. Randomized controlled study evaluating efficiency of low intensity transcranial direct current stimulation (tDCS) for dyspnea relief in mechanically ventilated COVID-19 patients in ICU: THe tDCS-DYSP-COVID Protocol. Front Med (Lausanne). (2020) 7:372. 10.3389/fmed.2020.00372 32671084 PMC7332773

[34] Fudim M Qadri YJ Ghadimi K MacLeod DB Molinger J Piccini JP. Implications for neuromodulation therapy to control inflammation and related organ dysfunction in COVID-19. J Cardiovasc Transl Res. (2020) 13:894–9. 10.1007/s12265-020-10031-6 32458400 PMC7250255

[35] Pilloni G Bikson M Badran BW George MS Kautz SA Okano AH. Update on the use of transcranial electrical brain stimulation to manage acute and chronic COVID-19 symptoms. Front Hum Neurosci. (2020) 14:595567. 10.3389/fnhum.2020.595567 33281589 PMC7689057

[36] Borovikova LV Ivanova S Zhang M Yang H Botchkina GI Watkins LR. Vagus nerve stimulation attenuates the systemic inflammatory response to endotoxin. Nature. (2000) 405:458–62. 10.1038/35013070 10839541

[37] Huston JM. The vagus nerve and the inflammatory reflex: wandering on a new treatment paradigm for systemic inflammation and sepsis. Surg Infect (Larchmt). (2012) 13:187–93. 10.1089/sur.2012.126 22913335

[38] Martelli D McKinley MJ McAllen RM. The cholinergic anti-inflammatory pathway: a critical review. Auton Neurosci. (2014) 182:65–9. 10.1016/j.autneu.2013.12.007 24411268

[39] Oke SL Tracey KJ. The inflammatory reflex and the role of complementary and alternative medical therapies. Ann N Y Acad Sci. (2009) 1172:172–80. 10.1196/annals.1393.013 19743552 PMC4533858

[40] Olofsson PS Katz DA Rosas-Ballina M Levine YA Ochani M Valdes-Ferrer SI. Alpha7 nicotinic acetylcholine receptor (alpha7nAChR) expression in bone marrow-derived non-T cells is required for the inflammatory reflex. Mol Med. (2012) 18:539–43. 10.2119/molmed.2011.00405 22183893 PMC3356417

[41] Pavlov VA Chavan SS Tracey KJ. Bioelectronic medicine: from preclinical studies on the inflammatory reflex to new approaches in disease diagnosis and treatment. Cold Spring Harb Perspect Med. (2020) 10:a034140. 10.1101/cshperspect.a034140 31138538 PMC7050582

[42] Pavlov VA Tracey KJ. The vagus nerve and the inflammatory reflex–linking immunity and metabolism. Nat Rev Endocrinol. (2012) 8:743–54. 10.1038/nrendo.2012.189 23169440 PMC4082307

[43] Tarnawski L Reardon C Caravaca AS Rosas-Ballina M Tusche MW Drake AR. Adenylyl cyclase 6 mediates inhibition of TNF in the inflammatory reflex. Front Immunol. (2018) 9:2648. 10.3389/fimmu.2018.02648 30538698 PMC6277584

[44] Tracey KJ. The inflammatory reflex. Nature. (2002) 420:853–9. 10.1038/nature01321 12490958

[45] Wu YJ Wang L Ji CF Gu SF Yin Q Zuo J. The role of alpha7nAChR-mediated cholinergic anti-inflammatory pathway in immune cells. Inflammation. (2021). 10.1007/s10753-020-01396-6. [Epub ahead of print]. 33405021

[46] Yin Q Wu YJ Pan S Wang DD Tao MQ Pei WY. Activation of cholinergic anti-inflammatory pathway in peripheral immune cells involved in therapeutic actions of alpha-mangostin on collagen-induced arthritis in rats. Drug Des Devel Ther. (2020) 14:1983–93. 10.2147/DDDT.S249865 32546965 PMC7250306

[47] Zi S Li J Liu L Liu F. Cholinergic anti-inflammatory pathway and its role in treatment of sepsis. Zhong Nan Da Xue Xue Bao Yi Xue Ban. (2020) 45:68–73. 10.11817/j.issn.1672-7347.2020.180651 32132300

[48] Hu J Liu S Ma T. Research progress of exploring the treatment of sepsis based on cholinergic anti-inflammatory pathway. Zhonghua Wei Zhong Bing Ji Jiu Yi Xue. (2021) 33:122–5. 10.3760/cma.j.cn121430-20200421-00318 33565416

[49] Lulic D Ahmadian A Baaj AA Benbadis SR Vale FL. Vagus nerve stimulation. Neurosurg Focus. (2009) 27:E5. 10.3171/2009.6.FOCUS09126 19722820

[50] Ansari S Chaudhri K Al Moutaery KA. Vagus nerve stimulation: indications and limitations. Acta Neurochir Suppl. (2007) 97:281–6. 10.1007/978-3-211-33081-4_31 17691314

[51] Henry TR. Therapeutic mechanisms of vagus nerve stimulation. Neurology. (2002) 59:S3–14. 10.1212/WNL.59.6_suppl_4.S3 12270962

[52] Berthoud HR Neuhuber WL. Functional and chemical anatomy of the afferent vagal system. Auton Neurosci. (2000) 85:1–17. 10.1016/S1566-0702(00)00215-0 11189015

[53] Rosas-Ballina M Ochani M Parrish WR Ochani K Harris YT Huston JM. Splenic nerve is required for cholinergic antiinflammatory pathway control of TNF in endotoxemia. Proc Natl Acad Sci USA. (2008) 105:11008–13. 10.1073/pnas.0803237105 18669662 PMC2504833

[54] Andersson U Tracey KJ. Neural reflexes in inflammation and immunity. J Exp Med. (2012) 209:1057–68. 10.1084/jem.20120571 22665702 PMC3371736

[55] Huston JM Ochani M Rosas-Ballina M Liao H Ochani K Pavlov VA. Splenectomy inactivates the cholinergic antiinflammatory pathway during lethal endotoxemia and polymicrobial sepsis. J Exp Med. (2006) 203:1623–8. 10.1084/jem.20052362 16785311 PMC2118357

[56] Meregnani J Clarencon D Vivier M Peinnequin A Mouret C Sinniger V. Anti-inflammatory effect of vagus nerve stimulation in a rat model of inflammatory bowel disease. Auton Neurosci. (2011) 160:82–9. 10.1016/j.autneu.2010.10.007 21071287

[57] van Maanen MA Lebre MC van der Poll T LaRosa GJ Elbaum D Vervoordeldonk MJ. Stimulation of nicotinic acetylcholine receptors attenuates collagen-induced arthritis in mice. Arthritis Rheum. (2009) 60:114–22. 10.1002/art.24177 19116908

[58] Koopman FA Chavan SS Miljko S Grazio S Sokolovic S Schuurman PR. Vagus nerve stimulation inhibits cytokine production and attenuates disease severity in rheumatoid arthritis. Proc Natl Acad Sci USA. (2016) 113:8284–9. 10.1073/pnas.1605635113 27382171 PMC4961187

[59] Yamakawa K Matsumoto N Imamura Y Muroya T Yamada T Nakagawa J. Electrical vagus nerve stimulation attenuates systemic inflammation and improves survival in a rat heatstroke model. PLoS ONE. (2013) 8:e56728. 10.1371/journal.pone.0056728 23424673 PMC3570456

[60] Meneses G Bautista M Florentino A Diaz G Acero G Besedovsky H. Electric stimulation of the vagus nerve reduced mouse neuroinflammation induced by lipopolysaccharide. J Inflamm (Lond). (2016) 13:33. 10.1186/s12950-016-0140-5 27807399 PMC5086408

[61] Kohoutova M Horak J Jarkovska D Martinkova V Tegl V Nalos L. Vagus nerve stimulation attenuates multiple organ dysfunction in resuscitated porcine progressive sepsis. Crit Care Med. (2019) 47:e461–9. 10.1097/CCM.0000000000003714 30908312

[62] Zuanetti G De Ferrari GM Priori SG Schwartz PJ. Protective effect of vagal stimulation on reperfusion arrhythmias in cats. Circ Res. (1987) 61:429–35. 10.1161/01.RES.61.3.429 3621502

[63] Ben-Menachem E. Vagus nerve stimulation, side effects, long-term safety. J Clin Neurophysiol. (2001) 18:415–8. 10.1097/00004691-200109000-00005 11709646

[64] Morris GL III Gloss D Buchhalter J Mack KJ Nickels K Harden C. Evidence-based guideline update: vagus nerve stimulation for the treatment of epilepsy: report of the guideline development subcommittee of the american academy of neurology. Epilepsy Curr. (2013) 13:297–303. 10.5698/1535-7597-13.6.297 24348133 PMC3854750

[65] Ryvlin P Gilliam FG Nguyen DK Colicchio G Iudice A Tinuper P. The long-term effect of vagus nerve stimulation on quality of life in patients with pharmacoresistant focal epilepsy: the PuLsE (Open Prospective Randomized Long-term Effectiveness) trial. Epilepsia. (2014) 55:893–900. 10.1111/epi.12611 24754318 PMC4283995

[66] Frangos E Ellrich J Komisaruk BR. Non-invasive access to the vagus nerve central projections via electrical stimulation of the external ear: fMRI evidence in humans. Brain Stimul. (2015) 8:624–36. 10.1016/j.brs.2014.11.018 25573069 PMC4458242

[67] Fonseca RC Bassi GS Brito CC Rosa LB David BA Araujo AM. Vagus nerve regulates the phagocytic and secretory activity of resident macrophages in the liver. Brain Behav Immun. (2019) 81:444–54. 10.1016/j.bbi.2019.06.041 31271871 PMC7826199

[68] Staats P Giannakopoulos G Blake J Liebler E Levy RM. The use of non-invasive vagus nerve stimulation to treat respiratory symptoms associated with COVID-19: a theoretical hypothesis and early clinical experience. Neuromodulation. (2020) 23:784–8. 10.1111/ner.13172 32342609 PMC7267613

[69] Boezaart AP Botha DA. Treatment of stage 3 COVID-19 with transcutaneous auricular vagus nerve stimulation drastically reduces interleukin-6 blood levels: a report on two cases. Neuromodulation. (2020) 24:166–7. 10.1111/ner.13293 33063409 PMC7675307

[70] Bonaz B Sinniger V Pellissier S. Targeting the cholinergic anti-inflammatory pathway with vagus nerve stimulation in patients with Covid-19? Bioelectron Med. (2020) 6:15. 10.1186/s42234-020-00051-7 32743022 PMC7387121

[71] Burger AM D'Agostini M. Response to “The use of non-invasive vagus nerve stimulation to treat respiratory symptoms associated with COVID-19: a theoretical hypothesis and early clinical experience”. Neuromodulation. (2020) 23:1042–3. 10.1111/ner.13253 32762067 PMC7436494

[72] Tornero C Vallejo R Cedeno D Orduna J Pastor E Belaouchi M. A prospective, randomized, controlled study assessing vagus nerve stimulation using the gammaCore(R)-Sapphire device for patients with moderate to severe CoViD-19 Respiratory Symptoms (SAVIOR): a structured summary of a study protocol for a randomised controlled trial. Trials. (2020) 21:576. 10.1186/s13063-020-04486-w 32586395 PMC7316576

